# Superior Alignment of Human iPSC-Osteoblasts Associated with Focal Adhesion Formation Stimulated by Oriented Collagen Scaffold

**DOI:** 10.3390/ijms22126232

**Published:** 2021-06-09

**Authors:** Ryosuke Ozasa, Aira Matsugaki, Tadaaki Matsuzaka, Takuya Ishimoto, Hui-Suk Yun, Takayoshi Nakano

**Affiliations:** 1 Division of Materials and Manufacturing Science, Graduate School of Engineering, Osaka University, Suita 565-0871, Japan; ozasa@mat.eng.osaka-u.ac.jp (R.O.); matsugaki@mat.eng.osaka-u.ac.jp (A.M.); tadaaki.matsuzaka@mat.eng.osaka-u.ac.jp (T.M.); ishimoto@mat.eng.osaka-u.ac.jp (T.I.); 2 Department of Advanced Biomaterials Research, Materials Processing Innovation Research Division, Korea Institute of Materials Science, Changwon 51508, Korea; yuni@kims.re.kr

**Keywords:** induced pluripotent stem cell, cell therapy, bone regenerative medicine, cell proliferation, cellular arrangement, osteoblast, focal adhesion

## Abstract

Human-induced pluripotent stem cells (hiPSCs) can be applied in patient-specific cell therapy to regenerate lost tissue or organ function. Anisotropic control of the structural organization in the newly generated bone matrix is pivotal for functional reconstruction during bone tissue regeneration. Recently, we revealed that hiPSC-derived osteoblasts (hiPSC-Obs) exhibit preferential alignment and organize in highly ordered bone matrices along a bone-mimetic collagen scaffold, indicating their critical role in regulating the unidirectional cellular arrangement, as well as the structural organization of regenerated bone tissue. However, it remains unclear how hiPSCs exhibit the cell properties required for oriented tissue construction. The present study aimed to characterize the properties of hiPSCs-Obs and those of their focal adhesions (FAs), which mediate the structural relationship between cells and the matrix. Our in vitro anisotropic cell culture system revealed the superior adhesion behavior of hiPSC-Obs, which exhibited accelerated cell proliferation and better cell alignment along the collagen axis compared to normal human osteoblasts. Notably, the oriented collagen scaffold stimulated FA formation along the scaffold collagen orientation. This is the first report of the superior cell adhesion behavior of hiPSC-Obs associated with the promotion of FA assembly along an anisotropic scaffold. These findings suggest a promising role for hiPSCs in enabling anisotropic bone microstructural regeneration.

## 1. Introduction

Stem cell-based therapies and research have attracted attention as sources of novel bone regeneration techniques in orthopedics. Current therapeutic options for bone disorders and defects include the application of bone marrow-derived mesenchymal stem cells (MSCs), embryonic stem cells (ESCs), and induced pluripotent stem cells (iPSCs). iPSCs, which are pluripotent cells reprogrammed from somatic cells [1,2], are a promising cell source for regenerative medicine, as they not only exhibit renewability and pluripotency [3,4,5], but also overcome the limited availability of autologous MSCs [6] and the ethical and immunological concerns related to ESCs [3,4]. At present, many studies have reported the differentiation of iPSCs into osteoblasts and the generation of bone substitutes under treatment with bioactive molecules. The basal osteogenic medium used contains fetal bovine serum (FBS), ascorbic acid, β-glycerophosphate, and dexamethasone [7]. Moreover, bone morphogenetic proteins (BMPs), calcium-regulating vitamin D_3_ [8], and members of the TGF-β family [9] have been reported to enhance osteogenic differentiation. The successive formation of calcified structures was induced by treatment with retinoic acid, both in vitro and in vivo [10]. Other approaches, based on the exogenous overexpression of the transcription factor Runx2 in iPSCs [11] or intracellular interaction with co-cultured primary bone cells [12], have also been applied to induce the differentiation of iPSCs into osteoblasts.

The controlled cell adhesion of human iPSCs (hiPSCs) and their differentiated derivatives is necessary for regenerating functional tissues and organs. In particular, the process of bone tissue reconstruction requires the specific alignment of adhering cells and subsequent oriented matrix formation [13,14]. In a recent study, we revealed the unique behavior of human iPSC-derived osteoblasts (hiPSC-Obs): these cells synthesize highly ordered bone matrices with their bodies; these matrices are oriented along the collagen molecule axis on a bone-mimetic collagen scaffold [15]. These findings indicate the great potential of hiPSC-Obs for the construction of cell-produced bone matrices with the desired alignment properties. However, the biological mechanisms that regulate the anisotropic cellular behavior of hiPSC-Obs remain unknown. The present study focused on comparing normal human osteoblasts (NHObs) and hiPSCs in terms of the regulation of cell alignment responses to an anisotropic scaffold. Cellular recognition of scaffold texture is mediated by multiprotein focal adhesions (FAs), which are the main cellular structures linking the intracellular cytoskeleton to the extracellular matrix [16]. FAs are believed to be associated with diverse cell functions, including cytoskeletal organization, gene expression, and cellular migration, via the transmission of external signals to intracellular actin fibers [17]. Moreover, our recent studies revealed that FAs play essential roles in determining the orientation of bone matrices [18,19].

This study aimed to characterize the properties of hiPSC-Obs, including cell proliferation and cell morphology, observe the number and shape of their FAs, and examine the relationship between cell properties and FAs, using NHObs as control. To this end, we converted hiPSCs into osteoblasts via stepwise differentiation and evaluated their cellular behavior on an anisotropic collagen scaffold [20]. We used human fibroblast-derived iPSCs, because fibroblasts are widely used as a source of iPSCs [21].

## 2. Results

### 2.1. Induction of Osteogenic Differentiation of hiPSCs

**Figure 1:**
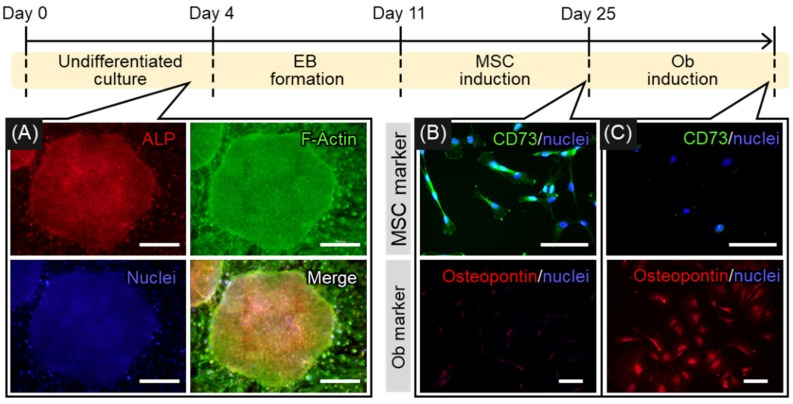
Stepwise differentiation of indifferent human induced pluripotent stem cells (hiPSCs) into osteoblasts (Obs) through mesenchymal stem cells (MSCs). (**A**) hiPSCs expressing ALP throughout the colony were observed for 4 days of culture on mitomycin C-inactivated SNL cells, representing an undifferentiated state of hiPSCs. (**B**) Positive expression of the MSC marker CD73 in cultured cells was observed on day 25, indicating that the cells had differentiated into MSCs. (**C**) Positive expression of osteopontin as an osteoblastic marker appeared in cultured cells on day 53; the cells were identified as osteoblasts (Obs). Day counting started from the first day of hiPSC culture. Scale bars: 100 µm.

**Figure 2:**
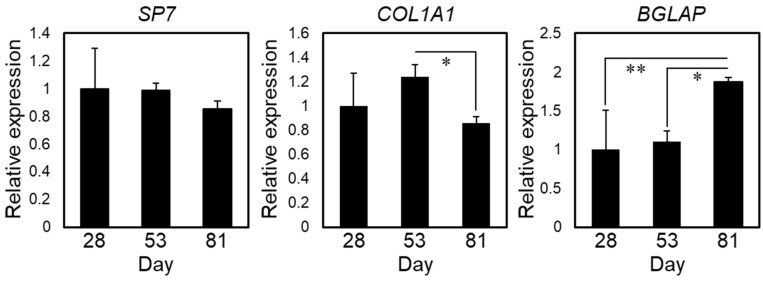
Expression of the osteoblastic markers *SP7*, *COL1A1*, and *BGLAP* over time. The data obtained from four biological replicates were averaged and are shown as the mean ± standard deviations (SDs). ** *p* < 0.01; * *p* < 0.05. Day counting started from the first day of hiPSC culture, as shown in Figure 1.

Serial changes in the differentiation of hiPSCs into osteoblasts are shown in Figure 1. hiPSCs were harvested on inactivated SNL feeder cells to maintain them in an undifferentiated state. The cells were undifferentiated for the first 4 days of culture, as demonstrated by their cobblestone-like appearance and the expression of alkaline phosphatase (ALP), a marker for pluripotent stem cells (Figure 1A). In addition, F-actin showed no notable location-dependent differences throughout the colony, which reflected the undifferentiated state of the hiPSCs. After the induction of MSCs through the formation of embryoid bodies (day 25), the cells turned positive for CD73, a specific marker of MSCs, and negative for osteoblastic markers (Figure 1B). This confirmed the differentiation of hiPSCs into MSCs. Subsequent culture in osteogenic inductive medium led to the differentiation of hiPSC-MSCs into bone-forming cells that express osteopontin, one of the osteoblastic markers (Figure 1C). Figure 2 shows gene expression relating to osteogenic differentiation over time. The expression of *COL1A1*, a marker for the early stage of osteogenic differentiation, peaked at day 53 and decreased at day 81, whereas the expression of *SP7* was unchanged. The expression of *BGLAP*, a marker for the late stage of osteogenic differentiation, peaked at day 81, indicating that osteoblastic maturation was initiated by long-term culture up to day 81.

### 2.2. Cellular Behavior on a Bone-Mimetic Collagen Scaffold

**Figure 3:**
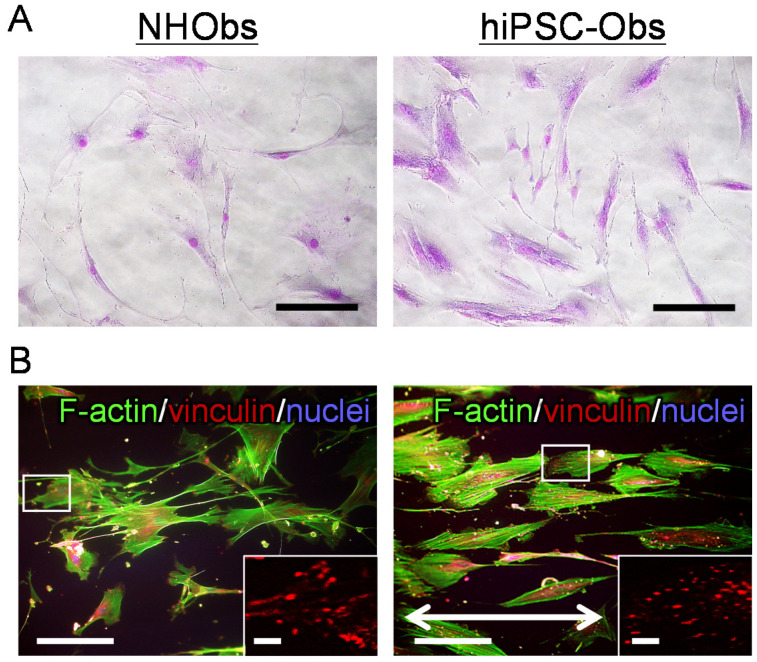
Observation of the cell behavior of human-induced pluripotent stem cell-derived osteoblasts (hiPSC-Obs) on a bone-mimetic collagen scaffold. (**A**) Non-uniform shape and random orientation of Giemsa-stained hiPSC-Obs and normal human osteoblasts (NHObs) on a cell culture plate. Scale bars: 100 µm. (**B**) Morphological changes of cells and focal adhesions (FAs) in hiPSC-Obs and NHObs on a collagen scaffold. The bidirectional arrows indicate the axis of collagen orientation on the scaffold. Green, F-actin; red, vinculin; blue, nuclei. Scale bars: 100 µm. The insets show magnified images of FAs of single cells. Scale bars: 10 μm.

**Figure 4:**
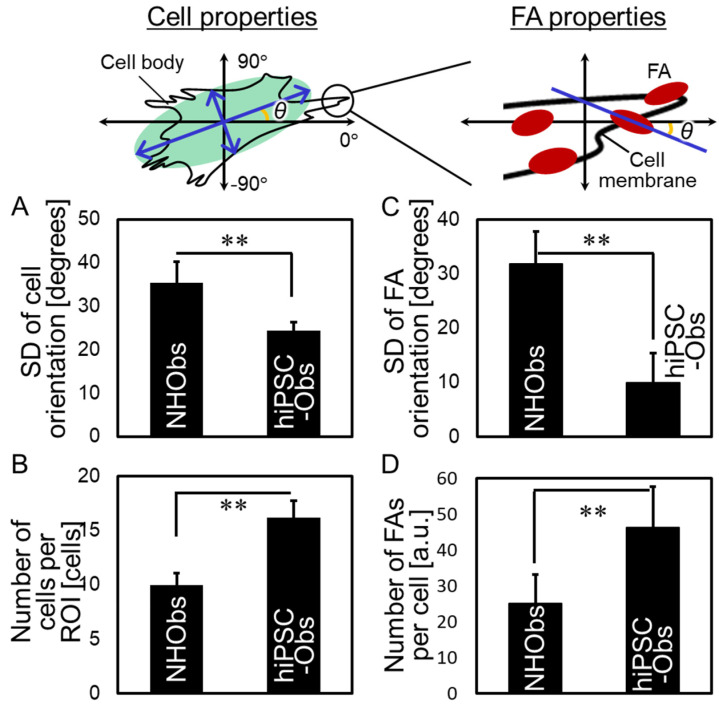
Estimation of the properties of cells and focal adhesions (FAs). The cell properties include: (**A**) the preferential cellular orientation, evaluated using the standard deviation (SD) of the cell orientation angle, and (**B**) cell proliferation, evaluated according to the cell number per region of interest (ROI). The FA properties include: (**C**) the preferential FA orientation, evaluated using the SD of the FA orientation angle, and (**D**) the number of FAs per cell. The data obtained from more than five biological replicates were averaged and are shown as the means ± SDs. ** *p* < 0.01.

The cellular properties and FAs of hiPSC-Obs were characterized through the analysis of cellular behavior on a bone-mimetic collagen scaffold (Figure 3 and Figure 4). On polystyrene culture plates, both hiPSC-Obs and NHObs exhibited non-uniform cell shapes and random orientations, as shown in Figure 3A. On the collagen scaffold, both types of osteoblasts showed elongated bodies and aligned predominantly along the extended axis of collagen fibers (Figure 3B). These results showed that the collagen scaffold promotes a preferential cellular orientation. Notably, hiPSC-Obs showed excellent responsiveness to the collagen scaffolds, exceeding that of the NHObs. In particular, the deviation of the cellular orientation angle with the extended axis of collagen was decreased (Figure 3B and Figure 4A), and cell proliferation was increased (Figure 4B) in hiPSC-Obs with respect to NHObs. In addition, the hiPSC-Obs exhibited an ordered array of FAs parallel to the cell orientation axis (Figure 3B and Figure 4C) and an increased number of FAs (Figure 3B and Figure 4D).

## 3. Discussion

**Figure 5:**
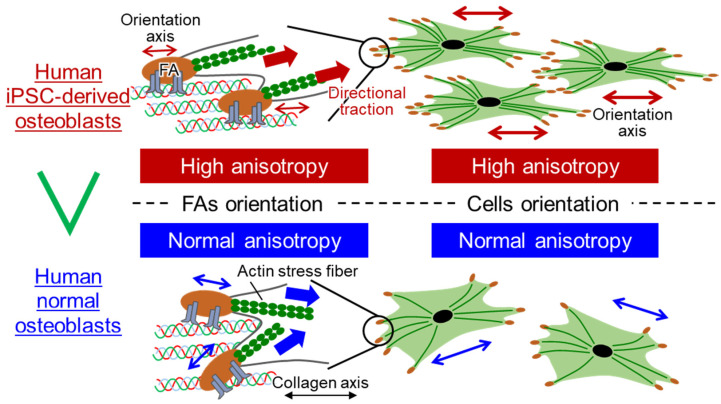
Schematic illustration of the relationship between cellular and FA properties in human induced pluripotent stem cell-derived osteoblasts (hiPSC-Obs) and normal human osteoblasts (NHObs). hiPSC-Obs exhibit a simultaneously enhanced orientation of FAs and cell reshaping along the collagen molecule substratum.

In the present study, we first characterized the superior properties of hiPSC-Obs and observed in particular: (i) a higher cell proliferation rate and degree of preferential orientation along the collagen scaffold than those of NHObs; (ii) a greater number of FAs and a higher degree of FA orientation along the collagen scaffold than those observed in NHObs; (iii) synchronous orientations of cell orientation and vinculin-positive FAs at the end of actin stress fibers. Based on these data, we demonstrated similar properties between cells (proliferation and orientation) and FAs (number and directionality), suggesting that the unique cellular properties of hiPSC-Obs depend on the formation of FAs upon recognition of the extracellular matrix substratum (Figure 5).

The interaction between cells and biomaterials is one of the crucial topics in the field of regenerative medicine [22,23,24,25]. Several in vitro studies have been conducted to artificially regulate cellular orientation using the geometry of the material surface [26], patterning [27,28], molecular arrays [14,15,29,30,31], and mechanical strain of the substrate [32] to control bone microstructure formation. Moreover, the orientation of natural osteoblasts in vivo has been reported to be associated with the structural properties of the collagen substratum embedded in the bone surface. In particular, Jones et al. observed that osteoblasts on the bone surface align parallel to the extended axis of the collagen fiber [33]. Baslé et al. pointed out that human Saos-2 osteoblast-like cells change their morphology in response to an anisotropic array of molecules and collagen-related amino acids, but not according to the shape of collagen fibers [34]. Therefore, the bone-mimetic scaffold composed of anisotropically ordered collagen molecules used in this study enabled the simulation of cellular behavior on the bone surface. The critical finding of this study is the possibility of accelerating the degree of cell alignment, which quantitatively determines the degree of orientation of the cell-derived matrix, using iPSC-derived osteoblasts. This suggests that the tissue microstructure formed during the regeneration process can be controlled on the basis of patient-specific cellular properties.

FAs function as sensors that allow cells to respond to matrices with various physical and molecular properties [16,18,19,35]. Hence, in this study, chemical cues such as cryptic RGD motifs [36] or GFOGE motifs [37] in oriented collagen fibers were considered to be sensed by FAs, thereby triggering cytoskeletal changes. The promotion of cell proliferation, together with the increased number of FAs (Figure 4B,D), was consistent with a previous study in which reduced FA formation inhibited cell proliferation via modulation of the FAK/RhoA-regulated mTORC1 and AMPK pathways [38]. Moreover, the maturation of FAs is one of the key regulatory mechanisms underlying the structural organization of regenerated tissue [18,19]. The present study’s findings demonstrate the promoted alignment of FAs in hiPSC-Obs, indicating the possibility of modulating tissue growth in a patient-specific, desired fashion.

Transmembrane integrin receptors are believed to play a central role in the regulation of FA formation and functionalization [39,40]. Integrins such as α_V_β_3_, α_5_β_1_, and α_6_β_1_ are commonly expressed in human osteoblasts [41] and fibroblasts [42,43,44]. In this sense, differences in FA properties between hiPSC-Obs derived from fibroblasts and NHObs might originate from individual differences between donors or the unique history of hiPSC-Obs, which first underwent reprogramming from fibroblasts by expressing *OCT3/4*, *SOX2*, *KLF4*, and *c-Myc*, and subsequently, osteogenic differentiation.

Interestingly, osteoblast alignment along collagen fibers is considered a universal cell behavior, independent of the animal species (primate or rodent). However, the degree of orientation differs notably between human-derived and mice-derived osteoblasts, as human-derived osteoblasts tend to exhibit a higher degree of cell alignment (*σ* = 24.3 ± 2.1 in hiPSC-Obs, *σ* = 35.3 ± 4.9 in NHObs; Figure 4B) than mice-derived osteoblasts (*σ* = 44.4 ± 2.3) [29], suggesting the possible advantage of the use of human-derived cells rather than rodent cells to produce highly oriented tissue structures. Another important application proposed in this study is the possibility of exploiting iPSCs for disease modeling due to their anisotropic cellular behavior. Several bone disorders in humans have limited treatment possibilities because of the inaccessibility of the affected bones. Genetic disorders, such as osteopetrosis [45] and osteogenesis imperfecta [46]; acquired disorders, including osteoporosis [47,48], cancer bone metastasis [49,50], and chronic kidney disease (CKD) [51]; regenerated bone [13,52]; and drug treatment [53,54] affect bone matrix microstructures, demonstrating the involvement of various biological mechanisms, including the autonomous or mutual activity of bone cells and their related biomolecules, in the formation of bone microstructure [55]. The biological mechanism underlying changes in the bone microstructure upon the emergence of bone disorders may be revealed in the future by an iPSC-based disease model composed of patient-derived hiPSC-Obs and the culture platform introduced in this study. Moreover, iPSC-based drug screening will accelerate the development of anti-osteoporotic drugs without adverse effects on bone microstructures.

Furthermore, the obtained hiPSC-Obs exhibited decreased expression of *COL1A1,* and conversely, increased expression of *BGLAP* after an adequate period of osteogenic induction. In general, *COL1A1* and *BGLAP* are used as markers for the early and late stage of osteogenic differentiation, respectively [56]. This implies that osteogenic differentiation proceeds over time, and that the hiPSC-Obs obtained in this study can be identified as mature osteoblasts. Moreover, osteocalcin (Ocn), the protein encoded by *BGLAP*, contains a gamma carboxyglutamate (Gla) domain, which mediates its affinity for the calcium ion of apatite [57,58,59]. The binding of Ocn to collagen was also observed in intra- and inter-fibrillar collagen spaces in vivo [60]. Ocn is synthesized specifically by mature osteoblasts, and 60–90% of Ocn is trapped in the bone matrix [61]. Notably, Ocn deficiency disrupts the epitaxial relationship between collagen and apatite in bones, resulting in reduced bone strength, while exerting no notable effects on the density or size of apatite crystallites [62]. These findings indicate a novel role of the Ocn molecule in adjusting the crystallographic orientation of the apatite *c*-axis parallel to the collagen fiber during the crystal nucleation of apatite occurring in the vicinity of the collagen hole zone. Therefore, the mechanism of osteogenic differentiation upon the upregulation of *BGLAP*, and thus, the increased production of the Ocn protein, is a promising research target in the field of stem cell-based therapy for bone tissues.

## 4. Materials and Methods

### 4.1. Culture of SNL Feeder Cells

SNL 76/7 cells were maintained on 0.1% gelatin-coated cell culture plates (IWAKI, Shizuoka, Japan) in Dulbecco’s Modified Eagle’s Medium (DMEM; Thermo Fisher Scientific, Waltham, MA, USA) containing 10% fetal bovine serum (FBS; Thermo Fisher Scientific), 100 U/mL penicillin (Thermo Fisher Scientific), 100 µg/mL streptomycin (Thermo Fisher Scientific), and 2 μM l-glutamine (Thermo Fisher Scientific) at 37 °C in a humidified atmosphere containing 5% CO_2_. After achieving 80–90% confluence, the cells were treated with a mitomycin C solution (Kyowa Kirin, Tokyo, Japan) for inactivation. Mitomycin C-inactivated SNL cells were used as feeder cells for hiPSCs.

### 4.2. Culture of hiPSCs

The 201B7 cell line was obtained from the Center for iPS Cell Research and Application, Kyoto University [2]. hiPSCs were maintained on mitomycin C-inactivated SNL feeder cells in Primate ES medium (ReproCELL Inc., Yokohama, Japan) supplemented with 4 ng/mL basic fibroblast growth factor (bFGF; FUJIFILM Wako Chemicals, Osaka, Japan) at 37 °C in a humidified atmosphere containing 5% CO_2_. The time intervals between the passages were optimized in this study. The culture medium was replaced daily. The undifferentiated state of the cells was confirmed by immunostaining of ALP [63], a marker for pluripotent stem cells, and F-actin. Decreased actin filament is considered a sign of the differentiated state of cells in iPSC colonies [64].

### 4.3. Osteogenic Differentiation of hiPSCs

In this study, osteogenic differentiation was induced through two steps: (i) induction of MSCs from hiPSCs (hiPSC-MSCs) and (ii) induction of osteogenic differentiation from hiPSC-MSCs. The differentiation protocol applied in this study was optimized based on previous studies [15,65,66]. To obtain embryoid bodies, hiPSCs were maintained in suspension for 7 days on low-attachment 96-well plates (IWAKI) in Primate ES medium supplemented with 4 ng/mL bFGF and 5 mM Y-27632. More than 50 embryoid bodies were clamped and plated onto 0.1% gelatin-coated cell culture plates in an MSC induction medium supplemented with α-modified Eagle’s medium (α-MEM; Thermo Fisher Scientific) containing 10% FBS, 200 mM l-glutamine, and 10 mM nonessential amino acids (NEAAs; Thermo Fisher Scientific), and cultured for up to 2 weeks to reach confluence at 37 °C in a humidified atmosphere containing 5% CO_2_. The culture medium was replaced twice per week. To induce osteogenic differentiation, the cells were maintained in α-MEM containing 10% FBS, 100 U/mL penicillin, 100 μg/mL streptomycin, 50 μg/mL ascorbic acid (Sigma-Aldrich, St. Louis, MO, USA), 10 mM α-glycerol phosphate (Tokyo Kasei Kogyo, Tokyo, Japan), and 50 nM dexamethasone (Thermo Fisher Scientific) at 37 °C in a humidified atmosphere containing 5% CO_2_. The culture period was optimized in the present study. The culture medium was replaced twice per week.

### 4.4. Culture of NHObs

The CC-2538 normal human osteoblast (NHOb) cell line (LONZA, Basel, Switzerland) was used as a control for hiPSC-Obs in the cell orientation experiment. According to the manufacturer’s instructions, the cells were maintained in an OGM^TM^ SingleQuots^TM^ medium (LONZA) at 37 °C in a humidified atmosphere containing 5% CO_2_ until confluence, after which they were passaged.

### 4.5. Gene Expression Analysis

Total RNA was extracted from the cultured cells using TRIzol reagent (Thermo Fisher Scientific). The expression levels of the osteogenic marker genes *SP7*, *COL1A1*, and *BGLAP* were assessed using quantitative polymerase chain reaction (PCR). The threshold number of cycles (Ct) was set within the exponential stage of the PCR reaction. The expression level of each target gene was determined by standardization with the expression level of *GAPDH*.

### 4.6. Immunostaining

Cultured cells were fixed with paraformaldehyde and then washed with PBST (PBS-Triton X100) containing normal goat serum (Thermo Fisher Scientific) or normal donkey serum (Thermo Fisher Scientific) at room temperature for 30 min to block non-specific antibody-binding sites. The cells were incubated with primary antibodies against alkaline phosphatase (Novus, Littleton, CO, USA), osteopontin (Rockland, Pottstown, PA, USA), CD73 (Santa Cruz Biotechnology, Dallas, TX, USA), and vinculin (Sigma-Aldrich). After washing with PBST, the cells were incubated with secondary antibodies, including Alexa Fluor^®®^ 488-conjugated donkey anti-goat IgG (Thermo Fisher Science), Alexa Fluor^®®^ 546-conjugated goat anti rabbit IgG (Thermo Fisher Science), and Alexa Fluor^®®^ 546-conjugated goat anti-mouse IgG (Thermo Fisher Scientific). F-actin was visualized using Alexa Fluor^®®^ 488-conjugated phalloidin (Thermo Fisher Scientific). Finally, the cells were washed with PBST and mounted with Prolong Gold antifade reagent with DAPI (Thermo Fisher Scientific). Fluorescent images were captured using a fluorescence microscope (Biozero; Keyence, Osaka, Japan) and processed using Adobe Photoshop 10.0.

### 4.7. Quantitative Analysis of the Properties of Cells and Focal Adhesions (FAs)

Before the cell orientation experiment, cells grown on culture plates were visualized by Giemsa staining (FUJIFILM Wako Chemicals) and observed using a fluorescent microscope. Next, the cells were cultured on bone-mimetic oriented collagen scaffolds for 3 days and immunostained as described above. The scaffolds used in this study were fabricated via a process described in a previous study [20]. The number of cells per region of interest (ROI) was counted manually. Cells and FAs were imaged using a fluorescent microscope; the images were processed using ImageJ software (NIH, Bethesda, MD, USA). The orientation angles (*θ*) of cells and FAs against the axis of scaffold collagen were analyzed using the Cell Profiler software (Broad Institute, Cambridge, MA, USA). More than five replicates were analyzed for each group.

### 4.8. Statistical Analysis

Statistical comparisons between two means were performed using a two-tailed unpaired Student’s *t*-test and *F*-test for homoscedasticity. Comparisons among three means were performed using one-way analysis of variance with post hoc Tukey HSD tests. Statistical significance was set at *p* < 0.05.

## 5. Conclusions

The biological events that regulate the anisotropic cellular behavior of human iPSC-derived osteoblasts (hiPSC-Obs) remain unknown. Herein, by developing an in vitro anisotropic culture model using an oriented collagen scaffold, we revealed, for the first time, that hiPSCs-Obs exhibit enhanced cell properties (proliferation and orientation) together with enhanced FA properties (number and directionality) compared to normal human osteoblasts. Importantly, the cell orientation exhibited a parallel relationship with FAs that extended along the scaffold collagen orientation. Our findings highlighted that FA formation stimulated by an oriented collagen scaffold coordinates the anisotropic cell behavior of hiPSC-Obs and suggested the promising role of hiPSCs in the generation of anisotropic bone microstructure.

## Data Availability

The data presented in this study are available on request from the corresponding author.
